# Provider perspectives on emergency department initiation of medication assisted treatment for alcohol use disorder

**DOI:** 10.1186/s12913-022-07862-1

**Published:** 2022-04-07

**Authors:** Thibault Philippine, Ethan Forsgren, Cassandra DeWitt, Inanna Carter, Maureen McCollough, Breena R. Taira

**Affiliations:** 1 David Geffen School of Medicine at UCLA, Los Angeles, CA USA; 2 Department of Emergency Medicine, Olive View-UCLA Medical Center, Sylmar, California USA

**Keywords:** Alcohol use disorder, Medication assisted treatment, Naltrexone, Implementation science, Behavior change wheel

## Abstract

**Background:**

Alcohol use disorder (AUD) is ubiquitous and its sequelae contribute to high levels of healthcare utilization, yet AUD remains undertreated. The ED encounter represents a missed opportunity to initiate medication assisted treatment (MAT) for patients with AUD. The aims of this study are to identify barriers and facilitators to the treatment of AUD in the ED, and to design interventions to address identified barriers.

**Methods:**

Using an implementation science approach based on the Behavior Change Wheel framework, we conducted qualitative interviews with staff to interrogate their perspectives on ED initiation of AUD treatment. Subjects included physicians, nurses, nurse practitioners, clinical social workers, and pharmacists. Interviews were thematically coded using both inductive and deductive approaches and constant comparative analysis. Themes were further categorized as relating to providers’ capabilities, opportunities, or motivations. Barriers were then mapped to corresponding intervention functions.

**Results:**

Facilitators at our institution included time allotted for continuing education, the availability of clinical social workers, and favorable opinions of MAT based on previous experiences implementing buprenorphine for opioid use disorder. Capability barriers included limited familiarity with naltrexone and difficulty determining which patients are candidates for therapy. Opportunity barriers included the limited supply of naltrexone and a lack of clarity as to who should introduce naltrexone and assess readiness for change. Motivation barriers included a sense of futility in treating patients with AUD and stigmas associated with alcohol use. Evidence-based interventions included multi-modal provider education, a standardized treatment algorithm and order set, selection of clinical champions, and clarification of roles among providers on the team.

**Conclusions:**

A large evidence-practice gap exists for the treatment of AUD with Naltrexone, and the ED visit is a missed opportunity for intervention. ED providers are optimistic about implementing AUD treatment in the ED but described many barriers, especially related to knowledge, clarification of roles, and stigma associated with AUD. Applying a formal implementation science approach guided by the Behavior Change Wheel allowed us to transform qualitative interview data into evidence-based interventions for the implementation of an ED-based program for the treatment of AUD.

**Supplementary Information:**

The online version contains supplementary material available at 10.1186/s12913-022-07862-1.

## Introduction

Alcohol Use Disorder (AUD) is a frequent diagnosis in the Emergency Department (ED) [1] and the third leading cause of preventable death in the United States [2]. An estimated 17.8% of adults experience AUD at some point during their lifetimes [3], leading to more than 95,000 annual deaths and an untold physical, financial, and emotional burden of disease [4]. Of those affected, it is estimated that less than 10% are treated with appropriate medication [5].

Despite more than 2.2 million annual ED encounters for chronic alcohol use [1], a national survey of emergency department directors reported that only 15% of departments had any policy in place to screen for AUD and only 9% had a policy to perform a brief intervention [6]; far fewer initiate pharmacotherapy for AUD. Instead, most patients who present to the ED with AUD are managed for acute complications and discharged to follow up with primary care [7].

The ED encounter represents a missed opportunity. Studies show that initiating medication-assisted treatment (MAT) for substance use disorders in the ED can significantly increase engagement in addiction treatment as well as decrease use of opioids and nicotine. Similar positive results are suggested for alcohol [8, 9]. Medications for treating AUD have historically included acamprosate, disulfiram, topiramate, gabapentin, and more recently naltrexone (NTX), though none are widely used in the ED. [6] We have chosen to focus on the initiation of NTX because it is a first line treatment for AUD, and is the expected practice for primary care in our health system [10]. Several trials and meta-analyses have demonstrated that NTX can help AUD patients in the outpatient setting be more accepting of treatment, maintain abstinence, and reduce heavy drinking days [11–20]. Efforts have been made to characterize and address barriers to prescribing NTX in the primary care setting, though no similar studies exist in the ED [21, 22]. Recent studies suggest that treatment with NTX can also reduce the rate of ED revisits and readmissions [23, 24]. Despite the evidence, NTX is infrequently prescribed from the ED, delineating a persistent evidence-practice gap.

One recent study demonstrated the feasibility of prescribing NTX from the ED, however reported limited follow-up and poor patient-centered efficacy for naltrexone among recipients [25]. This study demonstrates both the difficulty of introducing an intervention into a new clinical setting and raises questions as to why outcomes differed from the primary care literature.

Implementation science is defined as “the scientific study of methods to promote the systematic uptake of research findings and other evidence-based practices into routine practice, and, hence, to improve the quality and effectiveness of health services.” [26] Thus, our study adopts a formal implementation science approach in order to understand the potential of ED-based AUD treatment programs and to maximize the efficacy of the proposed intervention.

## Objective

The aim of this study is to identify and describe perceived barriers and facilitators to the initiation of ED-based treatment of AUD with NTX. We then apply the Behavior Change Wheel framework to map identified barriers to their appropriate intervention functions in order to design a theoretically-grounded, evidence-based program for the initiation of treatment of AUD in the ED.

## Methods

### Theoretical framework

The Behavior Change Wheel (BCW) is a framework used in Implementation Science to guide complex implementation design and has been used successfully in the healthcare setting [27, 28]. The framework acknowledges that at the core of any successful intervention is behavior change on the part of the stakeholders. In order to accomplish the tasks of understanding behavior and identifying appropriate intervention options, the BCW uses the COM-B model of behavior. The COM-B model assumes that a specified target behavior (B) is the result of the interaction between one’s physical and psychological capability (C), physical and social opportunity (O), and automatic and reflective motivation (M). Once identified, each of these behavioral elements can be mapped to relevant intervention functions on the BCW in order to design successful interventions. For a visual of the BCW and its COM-B components, we encourage the reader to visit the authors’ website [29].

Our target behavior was defined as the initiation of treatment of AUD from the ED, including prescribing and administering Naltrexone, counseling, and referring patients to further resources. The BCW framework proposes three stages of development for a behavior change intervention: Stage 1 focuses on understanding behavior; Stage 2 identifies intervention options; and Stage 3 identifies behavior change techniques and modes of delivery [28]. The BCW framework was chosen a priori to assist in mapping qualitative findings to candidate interventions and thus optimize the chances of successful implementation of AUD treatment in the ED.

### Study design

This is a qualitative study using an implementation science approach consisting of semi-structured interviews of Emergency Department staff to solicit their knowledge and perceptions of AUD treatment in the Emergency Department, and to inform the design of an ED-based AUD treatment program. Our team of clinicians and researchers met regularly throughout the process in order to review collected data, revise our interview guide, and integrate that evidence to shape the implementation plan. IRB approval was obtained from the Olive View-UCLA Medical Center IRB prior to commencement of any research.

### Provider interviews

The purpose of the provider interviews was to understand the barriers and facilitators present prior to the implementation in order to address each with targeted interventions. We developed an interview guide to explore staff perspectives on AUD, Naltrexone, and our proposed implementation. Questions focused on the challenges in caring for AUD patients, prior knowledge of NTX for AUD, concerns about the initiation of Naltrexone in the ED, and their preferences on the format for NTX-related training and education. Demographic information was collected including staff role, years of experience, and number of years at the institution. The interview guide developed for this study is provided as Additional File 1.

### Setting

Olive View-UCLA Medical Center is a public, safety-net Emergency Department with an academic affiliation, emergency medicine residency, and an annual census of approximately 60,000 patients. The ED has dedicated pharmacists and social workers available 24 h a day, 7 days a week, and a substance use navigator during business hours. A substance use navigator (SUN), under the California Bridge model of care, performs the role of a care coordinator for patients experiencing SUD [30]. As part of this model, a Bridge Clinic exists within our institution that allows patients with SUD to receive care until a primary care clinic with experience in treating SUD can be assigned.

### Participants and sampling

Purposive sampling was used to identify participants from each of the following categories: attending physicians, resident physicians, nurse practitioners (NPs), nurses (RNs), nursing supervisors, clinical social workers (CSWs), and pharmacists. Participants were approached face-to-face in the Emergency Department for in-person interviews, or via email for telephone interviews. Interviews continued in each of the categories until thematic saturation was reached.

### Procedure

In-person interviews were held in a private office or sub-waiting room. Telephone interviews were held at the subject’s convenience. All interviews were audio-recorded after the interviewer introduced themselves, made an attempt to establish rapport with the subject, explained the study and its goals, and obtained verbal consent. No field notes were made during interviews. Interviews were intended to range from 15 to 30 min.

### Researcher characteristics and reflexivity

The research team was comprised of emergency physicians and medical students. One emergency physician is an expert in SUD and is the co-director of the Bridge Clinic for opiate use disorder (MM), another is trained in research and implementation science (BRT), and a third has a prior background in harm reduction strategies (EF). During analysis, we reflected as a team on our positions and background and how they might impact the coding and interpretation of the data.

### Qualitative analysis

Qualitative data were anonymized and transcribed verbatim using a professional online transcription service. Transcripts were edited within a week of interview to ensure accuracy, then analyzed in Dedoose, a collaborative, cloud-based qualitative data analysis platform [31, 32]. We used both inductive and deductive approaches to analysis. We used open coding and constant comparative analysis to identify emergent patterns in the data and also applied the BCW COM-B model of behavior to identify barriers and facilitators to the target behavior. Combining these approaches to coding and analysis allowed us to link themes to the BCW and identify the corresponding intervention functions. The qualitative researchers (TP, EF, CD, BRT) met regularly during the coding process to discuss the coding, refine and consolidate identified themes, and resolve coding discrepancies. Member checking and deviant case analysis were used to enhance trustworthiness. All results are reported according to the COREQ standards for reporting of qualitative research and is included as additional file 2 [33].

## Results

### Interviews

A total of 25 interviews were conducted with various members of the emergency department, including attending physicians (*n* = 8), registered nurses (*n* = 5), resident physicians (*n* = 3), pharmacists (*n* = 3), social and clinical social workers (*n* = 3), nurse practitioners (*n* = 2), and a nurse supervisor (*n* = 1). Years of experience ranged from new RN graduates that had worked less than a year in the medical field, to attending physicians with up to 33 years of clinical practice. Due to COVID-19 distancing measures, on-site interviews were halted after the initial 9 interviews, and the remainder were completed virtually. No repeat interviews were conducted.

### Identifying barriers, facilitators, and intervention functions

Forty-two emergent themes were identified. Themes were consolidated as specific barriers or facilitators to implementation, and further categorized as relating to capability, opportunity, or motivation under the BCW COM-B model. The complete findings, relating the facilitators and barriers, their COM-B behavioral source, and their corresponding intervention function(s) can be found in Table 1 (Capability barriers), Table 2 (Opportunity barriers and facilitators), and Table 3 (Motivation barriers and facilitators).

**Table 1:** Capability-Related Barriers

BCW Source	Agent	Barrier/Facilitator	Quote	Intervention Function and Proposed Intervention
Capability (Psychological) [Barrier]	MD/NP/ RN	ED staff are unaware that NTX can be used to treat AUD.	“I just don’t think that a lot of us feel very comfortable with it because we don’t really have a lot of training in it. It’s not something covered in medical school and it’s not something that we currently do in the emergency department.” -Resident	Education: Lecture for residents during Grand Rounds. Presentation for faculty at faculty meeting.
				Education: Creation of posters describing NTX and treatment algorithm.
				Education: Presentation for nurses during morning huddles.
				Education: Establish champions among MDs and RNs that staff can approach with questions.
				Enablement: Creation of AUD & AWS order set that includes educational material about indications, dosing, side effects, and contraindications.
				Enablement: Creation of AUD & AWS guidelines.
Capability (Psychological) [Barrier]	MD/NP/ RN/CSW	ED staff unfamiliar with screening tools to identify AUD.	“Well, I think alcohol use disorder population is actually kind of a broad-- it’s a broad spectrum of disease. I think alcohol use disorder hits all groups, all populations. So there isn’t one specific population that will have it... It’s like, it’s really hard to have a uniform approach to it” -Attending	Training: Train providers on the use of AUDIT-C assessment.
			-“Are there specific scales or questionnaires used to diagnose alcohol use disorder in the emergency department?” -“I feel like that’s relatively limited in the emergency department. I think oftentimes we either catch on either their presenting complaints in one facet or another, as we just discussed, or the residents uncover it as part of the review of systems.”-Attending	Training: Create a script that staff can use when asking patients about their alcohol use.
Capability (Psychological) [Barrier]	MD/NP/ RN/CSW	ED staff find it difficult to identify a patient’s stage of change.	“Again, you have to admit it’s a gray area. They’re not either, ‘Oh, they’re ready now.’ Versus, ‘They’re not ready.’ There may be that gray area, that’s where I think you’re truly speaking to your patient and how to figure out--would he be receptive to it? -Attending	Training: Train staff in motivational interviewing to better identify patient’s stage of change.

Summary of barriers relating to the BCW model’s COM-B category of capability, with relevant staff identified, representative quotes, and proposed intervention functions

**Table 2:** Opportunity-Related Barriers and Facilitators

BCW Source	Agent	Barrier/Facilitator	Quote	Intervention Function and Proposed Intervention
Opportunity (Physical) [Barrier]	MD/NP	Pharmacy pre-approval for IM NTX discourages providers from prescribing.	“No, I don’t know what the restrictions or authorizations are. If it was a prior auth situation, that will be a substantial hurdle” -Attending	Enablement: Eliminate or streamline pre-approval form for IM NTX.
				Enablement: Remind providers the oral form of NTX is available if pre-approval for the IM form is unsuccessful.
Opportunity (Physical) [Barrier]	MD/NP/ RN/CSW	Lack of adequate discharge materials that are easily understood by patients.	“I would leave it as language is not the only barrier. Things like health literacy, world view and a lot of those things often go hand in hand, which makes it a triple whammy in terms of communicating with someone, particularly on a subject that I’m not truly an expert” -Attending	Enablement: Have patient educational materials appropriate for patients with low health literacy available in multiple languages (English, Spanish, Chinese, Tagalog).
			“I get phone calls sometimes and they tell me, “I don’t understand this instruction.” Even if the primary language is English. And I have to explain to them in layman’s terms, how they can understand.” -RN	
Opportunity (Physical) [Barrier]	MD/NP/ RN/CSW	Perception from staff that they are unable to effectively communicate with patients because of language barriers.	“The majority of my patients with alcohol use disorder speak Spanish, and I’m a certified bilingual but I’m not truly bilingual. Even with a translator, it’s a lot harder to have a subtle conversation either via a translator or in another language than it is with a native speaker. Querying somebody’s motivations by giving subtle instructions and consent is a lot harder. We keep things a bit simpler.” -Attending	Environmental Restructure: Hire more bilingual CSW and SUNs. Hire more in-person interpreters.
				Environmental Restructure: Recruit and retain more residents and physicians who are bilingual.
Opportunity (Physical) [Barrier]	Pharmacy	Limited supply of NTX available to the ED.	“Oh, yeah. We keep like absolutely nothing of the intramuscular, or very, very few, so if we actually did roll out our true protocol for patients, then we would have to really get in touch with the line management to get them to know we’re going to be rolling out this protocol and we need to have this many on hand, we’re expecting this much usage of this medication.” -Pharmacist	Environmental Restructure: Work with pharmacy to increase supply of IM NTX.
Opportunity (Physical) [Barrier]	MD/NP/ CSW	Unable to follow-up with provider who can continue NTX treatment.	“I guess my concern would be, like anything you start, is having the back-end follow it up. It’s easy start things but if we prescribe naltrexone, you say, “Here’s your prescription,” if they’re coming back in a week for refills or two weeks because there is poor substance use disorder assistance in the community.” -Attending	Environmental Restructuring: Utilize Substance Use Bridge Clinic to bridge AUD patient to next prescriber.
				Enablement: Compile list of inpatient and outpatient AUD treatment options.
				Environmental Restructuring: Perform warm hand-offs with organization that provides AUD treatment.
				Enablement: Increase number of Substance Use Navigators (SUNs) to help AUD patients access resources.
				Enablement: Encourage use of existing adjunct programs, such as community health workers, to help AUD patients access more care.
Opportunity (Physical) [Barrier]	MD/NP/ CSW	Patients often have unreliable access to a phone.	“I’m wasting my time on doing something, if no one else help me to do something, because I can’t get a hold of the patient. Giving resources, it works probably 10% of the time, if that, because maybe 10% of patients have a phone” -CSW	Enablement: Establish connection to cell phone distribution program
Opportunity (Physical) [Barrier]	CSW	Social workers feel they don’t have enough time with the patients.	“The time constraint is also an issue. Let’s say, for example, patients being interested in being referred for detox. That is a lengthy process because right now, the only detox referral we use is Tarzana Treatment Center. We have to get the information they want, and they’ll have the patient sign a consent and then fax it over. Then you have to wait for them to review it and then call you back and then to let you know if they have a bed or not. Most of the times they don’t even have a bed.” -CSW	Enablement: Ensure enough time is given for social workers to work with AUD patients. Refer to CSW early in the process.
				Enablement: Hire more SUNs.
Opportunity (Physical) [Barrier]	MD/NP	Cost of IM NTX is high.	“With the intramuscular injection, it’s ten times more expensive per month, so that’s one issue right there. And I’m not sure in terms of insurance how well that would work for some patients.” -Pharmacist	Enablement: Seek out cost-saving programs or subsidies offered by manufacturers.
Opportunity (Physical) [Barrier]	MD/NP/ RN/CSW	Sobering patients can remain in the ED for many hours, and the task of assessing for AUD can be missed prior to discharge.	“...you try to get them to the point where, clinically, they’re relatively sober in their clinical state and they’re safely able to discharge. Oftentimes I feel like the piece that’s missing is once you reassess them, [...] we would miss that opportunity to ask them, “Hey, now that I’m finally cognitively meeting you for the first time, are you interested in quitting?” -Attending	Environmental Restructure: Add screening for AUD to checklist of items to fulfill before discharge for patients who presented with intoxication.
Opportunity (Social) [Barrier]	MD/NP/ RN/CSW	Staff opposition to ED as source of long-term treatment.	“It’s easy to start things but if we prescribe naltrexone, you say, “Here’s your prescription,” and they’re coming back in a week for refills or two weeks because there is poor substance use disorder assistance in the community, then we’re becoming a continuity clinic, which-- no. It’s not how the ED is supposed to be” -Attending	Enablement: Strengthen communication between primary care and the emergency department.
				Modeling: Demonstrate a culture of providing all needed care in the ED, including initiating treatment and connection to care, in addition to immediate medical emergencies.
Opportunity (Social) [Barrier]	MD/NP/ RN/CSW	Roles and responsibilities not clearly defined, specifically, which staff will assess AUD and stage of change.	“It’s mostly by consult. Essentially, the doctor or the nurse contact the social worker if the patient is interested in resources... So they know we’re stopping by. Because a lot of times they consult us without asking the patient and we show up and they’re like, ‘Why are you here?’” -CSW	Environmental Restructuring: Clearly define and assign roles in the management algorithm in advance of the implementation.
Opportunity (Physical) [Facilitator]	CSW	Clinical Social Workers and Substance Use Navigators (SUNs) in the ED are available for referral most days 24/7.	“About four years ago [...] there was no substance abuse counselor, no social workers, no medical case workers at the ED. Comparing that from four years to about to now, what we have is we have 24/7 social worker and case workers as well as occasional substance abuse counselors 8–12 h a day.” -Attending	Enablement: Encourage consultation with on-site CSWs and SUNs, who can help with assessment of patients’ stage of change, treatment center referrals, phone acquisition, and connect the patient to other assistance programs.
Opportunity (Physical) [Facilitator]	Pharmacy	Pharmacists in the ED 24/7 for assistance with medications.	“Before there had only been one pharmacist in afternoon and then one at night for graveyard. And there were some hours where there were no ED pharmacists. They were able to change that though. So now we have 24-h coverage.” -Pharmacist	Enablement: Encourage discussion with pharmacists, who can help with details of Naltrexone prescription.
Opportunity (Physical) [Facilitator]	MD/NP	SUD Treatment Centers like Tarzana exist in the area for referral.	“I think we’re doing better. For example, for opioids use disorder we do have a substance abuse counselor. We have, for example, something set up with Tarzana Treatment Center, which I think even more have played a big part.” -NP	Enablement: Encourage connection to outside treatment centers and strengthen referral relationship and data sharing.
Opportunity (Physical) [Facilitator]	MD	Weekly Grand Rounds for MDs offers an established opportunity for new education and initiatives.	“I think that conference might be the best avenue just because you’ve got everyone, or more people there at once, who are more likely to be paying attention...” -Resident	Enablement: Leverage existing dedicated education time, i.e. departmental conference
Opportunity (Physical) [Facilitator]	RN	RN daily huddles, with longer huddles on weekends to address more complicated in-service topics.	“We always have daily huddles. So we can educate our staff that way.” -RN	Enablement: Leverage existing dedicated education time, i.e. RN huddles
			“During the weekends in the morning is when we have huddles that are lengthy. During the week the huddles are shorter, so we’ll do a lot of other stuff with short topics.” -RN	

Summary of barriers and facilitators relating to the BCW model’s COM-B category of opportunity, with relevant staff identified, representative quotes, and proposed intervention functions

**Table 3:** Motivation-Related Barriers and Facilitators

BCW Source	Agent	Barrier/Facilitator	Quote	Intervention Function and Proposed Intervention
Motivation (Automatic) [Barrier]	MD/NP	Perception that primary care providers are unfamiliar with Naltrexone for AUD.	“Anytime you have a service like this, the clinics [...] outside the hospital in different cities, basically, just refuse to deal with it and send everybody to the hospital... Whenever we establish one of these services, the clinic starts sending us those patients rather than referring through the usual mechanism” -Attending	Persuasion: Dispel ED provider misconceptions of primary care and reassure that AUD management exists in primary care.
Motivation (Automatic) [Barrier]	MD/NP/ RN/CSW	Staff feel powerless in helping AUD patients, and that attempting to treat them is futile.	“I guess I find it a challenge that I’m good at managing short-term, but I feel basically helpless in helping them get more definitive management of their problem.” -Attending	Persuasion: Use of encouraging examples and vignettes in conference. Liken to management of other chronic conditions, where success is expected to be gradual and is measured by progress.
				Modeling: Establish champions among MDs, Residents, and RNs that will model behavior and reiterate success stories.
Motivation (Automatic) [Barrier]	MD/NP/ RN/CSW	Staff bias against patients who use alcohol.	“There’s that coarse and vulgar term used, that goes ‘metabolize to freedom,’ where you try to get them to the point where, clinically, they’re relatively sober in their clinical state and they’re safely able to discharge.” -Attending	Modeling: Model alternative terminology, i.e. replace “metabolize to freedom” with “metabolize to screening.”
Motivation (Automatic) [Barrier]	MD/NP/ RN/CSW	Staff implicit bias of non-English speakers may be affecting their care.	“Our patients specifically, probably a language barrier. I think foremost. Just because we deal with a huge Hispanic population. For a lot of people, English isn’t their first language. So that’s going to be a big barrier. You know, Not quite understanding why, if they’re drinking a 12-pack a day for 10 years straight, and they decide to stop suddenly—they don’t quite understand why they’re shaking and anxious. So that’s one. I think that’s quite a big barrier” -RN	Modeling: Demonstrate cultural humility in approach to AUD management. Avoid “othering” of non-English speakers and AUD patients.
				Training: Regular implicit bias training for ED staff.
Motivation (Automatic) [Facilitator]	MD/NP/ RN/CSW	Positive opinion of medication assisted treatment and new initiatives among staff.	“More so, recently, I feel like there’s just more resources and potentially more knowledge and more advocates for these patients and I feel like there’s a higher rate of success in getting these patients to the next tangible step and not necessarily leaving them out into the void.” -Attending	Modeling: Continue to use the Bridge Clinic’s OUD management precedent as a model that can be used for AUD treatment.
				Enablement: Use positive opinion of opioid use disorder MAT to encourage the adoption of naltrexone as a treatment option for AUD.

Summary of barriers relating to the BCW model’s COM-B category of motivation, with relevant staff identified, representative quotes, and proposed intervention functions

### Capability (physical and psychological)

Capability barriers describe the physical and psychological capacity of staff to engage in the treatment of AUD. The most frequent barrier identified was a lack of familiarity with treatment options for AUD:

*“I just don't think that a lot of us feel very comfortable with it [Naltrexone] because we don't really have a lot of training in it. It’s not something covered in medical school and it's not something that we currently do in the emergency department.”*

ED staff also felt they lacked capacity to identify patients with AUD and to discern the patient’s interest in AUD treatment or stage of change. In order to address these gaps in knowledge, these barriers were mapped to educational, training, and enabling interventions to build providers’ capacity. Details of the educational, training, and enabling interventions can be found in Table 1.

### Opportunity (physical and social)

Opportunity barriers and facilitators describe factors external to providers that impede, prompt, or modify their treatment of AUD. Ambiguity in responsibilities between medical providers and clinical social workers created confusion about who was screening for AUD and assessing patients’ readiness to change:

*“It’s mostly by consult. Essentially, the doctor or the nurse contacts the social worker if the patient is interested in resources. [...] So they know we're stopping by. Because a lot of times they [the providers] consult us without asking the patient and we [clinical social workers] show up and they're like, ‘Why are you here?’”*

Many physicians and clinical social workers assumed the other was responsible for initiating a discussion on AUD with patients. The BCW suggests an environmental restructure to address this barrier, which can be fulfilled by clearly defining the roles of all staff with regards to AUD treatment, and increasing dialogue between providers and clinical social workers. Some raised concern about the role of EDs in providing care that would require long-term management:

*“It’s easy to start things but if we prescribe naltrexone, you say, ‘Here's your prescription,’ and they're coming back in a week for refills or two weeks because there is poor substance use disorder assistance in the community, then we're becoming a continuity clinic, which– no. It's not how the ED is supposed to be.”*

Although many expressed support for MAT and were enthusiastic about the possibility of offering more for AUD patients from the ED, others were hesitant about the ED becoming a source of long-term management for SUD. To address this barrier, the BCW suggests modeling and enablement interventions. Selecting champions to model the behavior of NTX prescription to demonstrate the potential of NTX is one potential remedy. Better processes that enable transition to the primary care setting by coordinating AUD treatment and sharing educational materials with primary care colleagues are additional options.

Lack of resources and options for follow-up was an additional concern. The specific lack of capacity at SUD treatment centers, as well as insufficient social support for patients such as reliable housing, food were mentioned. Access to a phone was considered crucial:

*"I'm wasting my time on doing something, if no one else help me to do something, because I can't get a hold of the patient. Giving resources, it works probably 10% of the time, if that, because maybe 10% of patients have a phone"*

Enablement in this case might entail bringing phone access programs into medical centers.

Interviews with pharmacists revealed multiple logistical barriers. The hospital’s mandatory pre-approval form (referred to as prior authorization in interviews) for ordering IM NTX was problematic. Providers agreed that having to complete a pre-approval form would be a major disincentive to ordering IM NTX. Additionally, pharmacy staff considered the cost of the intramuscular form of Naltrexone prohibitive.

*“With the intramuscular injection, it’s ten times more expensive per month, so that’s one issue right there. And I’m not sure in terms of insurance how well that would work for some patients.”*

Modifying costs and streamlining processes are potential solutions. We sought out pharmaceutical company cost reduction programs and recommended removing the pre-approval form in addition to focusing the intervention design on the oral version of naltrexone, which was significantly more affordable and did not require authorization.

Facilitators are themes or elements that are already in place at our ED that may contribute to care for AUD patients. Identified facilitators were mostly components of opportunity and were factors external to the individual provider. They include access to regularly scheduled continuing education for physicians and nurses, the availability of ED clinical social workers, pharmacists, and substance use navigators, and the proximity of the hospital to SUD treatment centers.

### Motivation (automatic and reflective)

Motivational barriers and facilitators describe the thought processes and emotional responses that providers associate with the treatment of AUD. We found that providers’ limitations in their perceived or real capabilities and opportunities (such as those described above) triggered emotional reactions of powerlessness when treating patients with chronic AUD:

*“I have my streamlined protocol but I guess I'm aware that my streamlined protocol works for me for short-term management of severe withdrawal symptoms, but doesn't address the underlying problem. I guess I find it a challenge that I'm good at managing short-term, but I feel basically helpless in helping them get more definitive management of their problem.”*

This emotion was reinforced by an experience of futility and a stigma against patients with AUD, assuming that such patients either did not want to quit or were unable:

*“You know, it’s a loop. We see it all the time. They get out of the ER, they go to 7-Eleven, they buy the alcohol at 7-Eleven, they go outside, they drink it, they get drunk, 7-Eleven calls 911, they come back to the ER, and then from the ER, they go back to 7-Eleven.”*

Furthermore, providers expressed fear that if the ED started offering more AUD services, that primary care clinics would start referring more patients with AUD to the ED, creating an influx of “difficult patients”:

*“Anytime you have a service like this, the clinics [...] outside the hospital in different cities, basically, just refuse to deal with it and send everybody to the hospital.”*

The BCW mapped these barriers to intervention functions of modeling, enablement, and persuasion. We will augment trainings to educate ED providers about the capability of our primary care providers to treat AUD in the clinics, draw analogies to how AUD treatment is similar to other chronic diseases, like diabetes, and celebrate examples of providers and patients who made progress through using the new treatment and referral pathways.

We found that providers had developed mental heuristics to summarize many of these emotions, communicate them to each other, and avoid frustration/complexity.

*“There’s that coarse and vulgar term used that goes ‘metabolize to freedom,’ where you try to get them to the point where, clinically, they're relatively sober in their clinical state and they’re safely able to discharge. Oftentimes I feel like the piece that's missing is, once you reassess them, they can eat, they could walk, they’re safe to be discharged, I feel like oftentimes we miss that opportunity to ask them, "Hey, now that I’m finally cognitively meeting you for the first time, are you interested in quitting?"*

We propose modeling using alternative catch-phrases such as “metabolize to screening” to emphasize the need to reevaluate intoxicated patients once they have sobered, including screening for AUD and assessing patient interest in treatment. A significant facilitator related to motivation is the pre-existing culture that views MAT positively, likely due to an existing successful program for opiate use disorder.

## Discussion

Several limitations are to be considered for this study. This is a single center study in a public emergency department. The demographics of the population served may limit generalizability of the findings. One key stakeholder group that is missing from the current study is the patients. Because of COVID restrictions, we have not yet been able to incorporate the patient perspective, however, this is an immediate next step. We also have a well-established, ED-based OUD treatment program. Its presence and success may lead providers to be more open-minded about the implementation of a similar program for AUD. This data and the use of the BCW suggests the intervention functions identified and the interventions developed will facilitate a successful implementation, however, the true success of the implementation will be seen in the next stage of this research when we measure process and patient-centered outcomes.

Although medication assisted therapy for AUD is effective [19], a large evidence-practice gap exists. Even in a group of medically insured patients with access to care, rates of use of pharmacotherapy for AUD are reported to be less than 10% [34]. Efforts have been made to increase AUD treatment in the primary care setting, including increasing the uptake of AUD treatment with naltrexone, through a hub and provider model [35]. However, a number of barriers have been described in the primary care setting, including a lack of knowledge and experience, cost, beliefs that medications cannot replace specialty addiction treatment, and alcohol-related stigma [21, 22].

As the evidence-practice gap persists, health systems have looked for other settings in which to target patients with AUD for the initiation of MAT. Recent evidence suggests that the initiation of MAT for AUD at the point of inpatient discharge not only increased the rate of MAT prescription but also lead to a decrease in unexpected 30 days ED revisits for patients with AUD [23, 24]. Alcohol related visits to the ED are common, increasing, and cost the U.S. health system billions of dollars per year [1, 36].

Past ED-based initiatives assessed the paradigm of SBIRT (Screening, Brief Intervention and Referral to Treatment) [37]. SBIRT is widely used as the American College of Surgeons required it for hospitals to maintain their trauma center designation [38]. Recent evidence suggests, however, that SBIRT is insufficient to improve outcomes in those with AUD [39]. SBI alone has not been shown to be effective for severe unhealthy alcohol use and the evidence for those with some unhealthy alcohol use has been inconsistent [39]. Screening and referral from the ED does not appear to be enough. With SBIRT (adding the Referral to Treatment), a major barrier is the failure to connect with the specialty clinic for treatment, and thus failure to initiate meaningful treatment [40].

The emergency department visit represents an opportune window to *initiate* AUD treatment because the ED visit is frequently the only point of contact with the health system for patients with SUD [41]. The ED visit is increasingly recognized as an important point for intervention for patients with SUD [41], and the ED has been shown as an effective venue for MAT programs focused on the treatment of opiate use disorder [8]. Given early successes of MAT for AUD treatment at hospital discharge and MAT for Opiate Use Disorder (OUD) in the ED, the logical extension will be to use the ED visit as a site for MAT initiation for AUD. A recent systematic review found, however, that ED-specific research into MAT for AUD is absent [7].

In initiating a new intervention in a busy and complex setting such as an Emergency Department, a formal approach to intervention design may increase the chances of successful uptake of the intervention. Interventions that are based in behavioral theory are more effective than those that lack a strong theoretical basis [42–44]. The BCW has been used in the past for stepwise assessment, stakeholder input, and intervention design that is rooted in behavioral theory [45, 46]. We chose the BCW because of its focus on provider behavior change. The BCW aided us in identifying specific intervention functions needed to change role specific provider behavior with regard to AUD treatment in the ED.

Much as in primary care, we found that the major barriers to the implementation of an AUD program in the Emergency Department included lack of knowledge and stigma associated with AUD both in terms of patients disclosing their use and also in terms of providers’ perceptions of alcohol use. Factual knowledge is easier to address, particularly in a department with a residency training program and a weekly academic conference. Other environments may not have such readily available mechanisms for group education. Stigma, however, presents a more difficult challenge. The stigma associated with patients with AUD and the discomfort with patient-provider communication is present in our ED despite the presence of an ongoing and successful MAT program for OUD. Although the OUD program was cited by participants as analogous to MAT for AUD and a potential facilitator for AUD program implementation, stigma associated with AUD is still prevalent. Individuals with AUD are less likely to engage with services if they perceive highly stigmatized views of alcoholism [47]. Reducing stigma amongst staff about patients with AUD will be a critical part of implementation. Well planned educational interventions can reduce levels of stigma surrounding AUD [48]. For those in settings where no prior OUD program exists, the stigma is likely to be even stronger.

In addition to the stigma related to AUD, we also heard generalizations about patients with limited English proficiency, who were assumed to have lesser knowledge of AUD when compared to English speakers. While health literacy levels and knowledge of substance use disorder is expected to vary between patients, the assumption that limited English proficiency patients are unaware of their alcohol use disorder signals underlying implicit bias on the part of the provider. Implicit bias is a much larger issue as the “othering” of patients has implications for all patient care. The explicit finding in this context emphasizes the need to include cultural humility as a core theme within the AUD education as well as the need for continued emphasis overall.

## Conclusion

In summary, there is a large evidence-practice gap for the treatment of AUD with Naltrexone. The ED visit is a missed opportunity for intervention in the care of AUD patients and initiation of Naltrexone. ED providers are optimistic about implementing AUD treatment in the ED but described many barriers, especially related to knowledge, clarification of roles, and stigma associated with AUD. Applying a formal implementation science approach guided by the Behavior Change Wheel allowed us to transform qualitative interview data into evidence-based interventions for the implementation of an ED-based program for the treatment of AUD.

## Supplementary Information

**Additional file 1.**

**Additional file 2.**

## Data Availability

Not applicable.

## Abbreviations

AUD: Alcohol Use Disorder
ED: Emergency Department
MAT: Medication-Assisted Treatment
BCW: Behavior Change Wheel
NTX: Naltrexone
SUN: Substance-Use Navigator
CSW: Clinical Social Worker
SUD: Substance Use Disorder
IM: Intramuscular
OUD: Opioid Use Disorder
