# Supplementary material for: Provider perspectives on emergency department initiation of medication assisted treatment for alcohol use disorder
Source: BMC Health Serv Res. 2022 Apr 7;22:456. doi: 10.1186/s12913-022-07862-1 (PMC8988541; doi:10.1186/s12913-022-07862-1)
Supplement: Supplementary file 1 — Additional file 1. [file 12913_2022_7862_MOESM1_ESM.docx]

Provider Perspectives on Emergency Department Initiation of Medication Assisted Treatment for Alcohol Use Disorder

**Interview Guide**

Date:

*As you may know, alcohol use disorder is the most prevalent substance use disorder among our patients. Our emergency department, together with other county emergency departments and UCLA, are considering training staff to prescribe and administer a medication called naltrexone to help reduce the harms of alcohol use. We are interviewing ED providers as part of a study to understand the baseline knowledge of treatment options and challenges that providers face when treating patients with AUD in the ED. If you agree to participate, you will be asked several questions about your knowledge and opinions on patients with Alcohol Use disorder and treatment with Naltrexone. The interview will last about 15minutes. You will be recorded, participation is voluntary and your responses will be anonymous. You may stop the interview at any time or skip questions you feel uncomfortable with. Your responses will help us to develop educational materials and further ED based programs related to AUD.*

*Do you agree to participate? YES/NO*

*If yes, record consent and proceed. If no, thank them for their time and record a refusal in the log.*

**Demographics**

***1.* *What is your role in the Emergency Department?***

***2.* *How long have you worked in health care?***

**AUD**

***1.*** ***What do you find challenging about taking care of patients with Alcohol Use Disorder?***

2. ***How do these challenges compare to treating patients with other types of substance use disorders?***

***Naltrexone***

***1.*** ***What do you know about naltrexone being used for alcohol use disorder?***

***2.*** ***Do you think patients will be interested in using naltrexone?***

***3.*** ***Do you foresee any difficulties with prescribing or administering naltrexone to patients in the emergency department?***

***4.*** ***What are your concerns about the use of naltrexone for patients with alcohol use disorder?***

***5.*** ***What kind of additional information or training about naltrexone would be helpful to you?***

***6.*** ***Are there additional things that you think we should be doing to help patients with alcohol use disorder?***

***7.*** ***Is there anything else you’d like to tell us?***

*Thank you for talking with us. Your answers are really valuable as we decide the role of Naltrexone treatment in our Emergency Department.*

***Addendum: Additional Questions for Consideration***

*How do you see the role of your position ( attendings, nurses, social workers, pharmacists etc) in implementation of naltrexone for AUD in the emergency department?*

*Open ended questions:*

*Walk me through last time you had a patient with alcohol use disorder?*

*At time of presentation:*

*How did you identify they had AUD?*

*How do you assess the stages of change?*

*What happened with this patient during their time in the ED? At time of discharge?*

*What do you typically do at time of discharge for patients with AUD?*

*What steps have to happen before a patient with AUD is discharged?*

*How can you support Naltrexone prescription at discharge (Pills or IM injection)?*

*Follow up questions: Did that worry you? How did you feel about that decision/moment/challenge? What were your concerns? How would you change that encounter? Walk me through that?*

*Questions related to MAT:*

*What are your thoughts on medication assisted treatment for substance use disorder such as buprenorphine, suboxone, methadone, etc…?*

*What are your thoughts on long term medication assisted treatment?*

*Walk me through a time you prescribed buprenorphine?*

*If you haven’t prescribed buprenoprhine, why haven’t you?*

*Any experience calling the on-call prescriber for X waiver?*

*Are you interested in an X waiver?*

*If interested in X waiver, but haven’t got it, why not?*

***Barriers:***

*From your perspective is there anything else that you think is a barrier for patients with this intervention?*

*Have you experienced a time where a language barrier was present when treating a patient with AUD? If so how did this change the encounter?*
