# Supplementary material for: Provider perspectives on emergency department initiation of medication assisted treatment for alcohol use disorder
Source: BMC Health Serv Res. 2022 Apr 7;22:456. doi: 10.1186/s12913-022-07862-1 (PMC8988541; doi:10.1186/s12913-022-07862-1)
Supplement: Supplementary file 2 — Additional file 2. [file 12913_2022_7862_MOESM2_ESM.docx]

Provider Perspectives on Emergency Department Initiation of Medication Assisted Treatment for Alcohol Use Disorder

**COREQ Checklist**

| **No. and Item**  Personal Characteristics | **Location** | **Guide Question/Description** |
| --- | --- | --- |
| 1.  Interviewer | *Declarations, Authors’ Contributions* | Which author/s conducted the interview or focus group? |
| 2. Credentials | *Title Page* | What were the researcher's credentials? *E.g. PhD, MD* |
| 3. Occupation | *Methods, Researcher Characteristics and Reflexivity* | What was their occupation at the time of the study? |
| 4. Gender | *Declarations, Authors’ Contributions* | Was the researcher male or female? |
| 5. Experience and training | *Methods, Researcher Characteristics and Reflexivity* | What experience or training did the researcher have? |
| Relationship with participants |  |  |
| 6. Relationship established | *Methods, Procedure* | Was a relationship established prior to study commencement? |
| 7. Participant knowledge of the interviewer | *Additional Files 1, Interview Guide* | What did the participants know about the researcher? e*.g. personal goals, reasons for doing the research* |
| 8. Interviewer characteristics | *Methods, Researcher Characteristics and Reflexivity* | What characteristics were reported about the interviewer/facilitator? e.g. *Bias, assumptions, reasons and interests in the research topic* |
| **Domain 2: study design** |  |  |
| Theoretical framework |  |  |
| 9. Methodological orientation and Theory | *Methods, Theoretical Framework* | What methodological orientation was stated to underpin the study? *e.g. grounded theory, discourse analysis, ethnography, phenomenology, content analysis* |
| Participant selection |  |  |
| 10. Sampling | *Methods, Participants and Sampling* | How were participants selected? *e.g. purposive, convenience, consecutive, snowball* |
| 11. Method of approach | *Methods, Participants and Sampling* | How were participants approached? e*.g. face-to-face, telephone, mail, email* |
| 12. Sample size | *Results, Interviews* | How many participants were in the study? |
| 13. Non-participation | *N/A* | How many people refused to participate or dropped out? Reasons? |
| Setting |  |  |
| 14. Setting of data collection | *Methods, Procedures* | Where was the data collected? e*.g. home, clinic, workplace* |
| 15. Presence of non-participants | *Methods, Procedures* | Was anyone else present besides the participants and researchers? |
| 16. Description of sample | *Results, Interviews* | What are the important characteristics of the sample? *e.g. demographic data, date* |
| Data collection |  |  |
| 17. Interview guide | *Methods, Study Design; Additional Files 1, Interview Guide* | Were questions, prompts, guides provided by the authors? Was it pilot tested? |
| 18. Repeat interviews | *Results, Interviews* | Were repeat interviews carried out? If yes, how many? |
| 19. Audio/visual recording | *Methods, Procedure* | Did the research use audio or visual recording to collect the data? |
| 20. Field notes | *Methods, Procedure* | Were field notes made during and/or after the interview or focus group? |
| 21. Duration | *Methods, Procedure* | What was the duration of the interviews or focus group? |
| 22. Data saturation | *Methods, Participants and Sampling* | Was data saturation discussed? |
| 23. Transcripts returned | *N/A* | Were transcripts returned to participants for comment and/or correction? |
| **Domain 3: analysis and findings** |  |  |
| Data analysis |  |  |
| 24. Number of data coders | *Declarations, Authors’ Contributions* | How many data coders coded the data? |
| 25. Description of the coding tree | *Results, Identifying Barriers, Facilitators, and Intervention Functions* | Did authors provide a description of the coding tree? |
| 26. Derivation of themes | *Methods, Qualitative Analysis* | Were themes identified in advance or derived from the data? |
| 27. Software | *Methods, Qualitative Analysis* | What software, if applicable, was used to manage the data? |
| 28. Participant checking | *Methods, Qualitative Analysis* | Did participants provide feedback on the findings? |
| Reporting |  |  |
| 29. Quotations presented | *Results, throughout* | Were participant quotations presented to illustrate the themes / findings? Was each quotation identified? e*.g. participant number* |
| 30. Data and findings consistent | *Results, throughout; Tables 1, 2, 3* | Was there consistency between the data presented and the findings? |
| 31. Clarity of major themes | *Results, throughout; Tables 1, 2, 3* | Were major themes clearly presented in the findings? |
| 32. Clarity of minor themes | *Tables 1, 2, 3; Discussion, last paragraph (p.17)* | Is there a description of diverse cases or discussion of minor themes? |
